# Hyperthymic affective temperament and hypertension are independent determinants of serum brain-derived neurotrophic factor level

**DOI:** 10.1186/s12991-016-0104-4

**Published:** 2016-07-29

**Authors:** János Nemcsik, Andrea László, Lilla Lénárt, Dániel Eörsi, Péter Torzsa, Beáta Kőrösi, Orsolya Cseprekál, András Tislér, Ádám Tabák, Xenia Gonda, Zoltán Rihmer, Judit Hodrea, Zsófia Nemcsik-Bencze, Andrea Fekete

**Affiliations:** 1 Department of Family Medicine, Semmelweis University Budapest, Budapest, Hungary; 2 Health Service of Zugló (ZESZ), Budapest, Hungary; 3 Ist Department of Pediatrics, Semmelweis University, Budapest, Hungary; 4 MTA-SE “Lendület” Diabetes Research Group Budapest, Budapest, Hungary; 5 Department of Transplantation and Surgery, Semmelweis University, Budapest, Hungary; 6 Ist Department of Internal Medicine, Semmelweis University, Budapest, Hungary; 7 Department of Epidemiology and Public Health, University College London, London, UK; 8 Department of Pharmacodynamics, Semmelweis University, Budapest, Hungary; 9 Department of Psychiatry and Psychotherapy, Semmelweis University, Budapest, Hungary; 10 National Institute of Psychiatry and Addictions, Budapest, Hungary; 11 Magnetic Resonance Imaging Research Center, Semmelweis University, Budapest, Hungary

**Keywords:** Brain-derived neurotrophic factor, Hypertension, Affective temperaments, Arterial stiffness

## Abstract

**Background:**

Brain-derived neurotrophic factor (BDNF) has neuroprotective, proangiogenic and myogenic effects and, therefore, possibly acts as a psychosomatic mediator. Here, we measured serum BDNF (seBDNF) level in hypertensive patients (HT) and healthy controls (CONT) and its relation to affective temperaments, depression and anxiety scales, and arterial stiffness parameters.

**Methods:**

In this cross-sectional study, affective temperaments, anxiety, and depression were studied with questionnaires (TEMPS-A, HAM-A, and BDI, respectively). SeBDNF level and routine laboratory parameters were measured as well. Arterial stiffness was evaluated with a tonometric method.

**Results:**

Allover, 151 HT, and 32 CONT subjects were involved in the study. SeBDNF level was significantly higher in HT compared to CONT (24880 ± 8279 vs 21202.6 ± 6045.5 pg/mL, *p* < 0.05). In the final model of regression analysis, hyperthymic temperament score (*Beta* = 405.8, *p* = 0.004) and the presence of hypertension (*Beta* = 6121.2, *p* = 0.001) were independent determinants of seBDNF. In interaction analysis, it was found that in HT, a unit increase in hyperthymic score was associated with a 533.3 (95 %CI 241.3–825.3) pg/mL higher seBDNF. This interaction was missing in CONT.

**Conclusions:**

Our results suggest a complex psychosomatic involvement of BDNF in the pathophysiology of hypertension, where hyperthymic affective temperament may have a protective role. BDNF is not likely to have an effect on large arteries.

## Background

Brain-derived neurotrophic factor (BDNF) is a member of the neurotrophic factor family, playing a central role in the regulation of neuronal growth, maintenance, and survival [1]. Its involvement in psychiatric conditions is well described and was confirmed by a meta-analysis, as in major depressive disorder, the decreased serum BDNF (seBDNF) level was elevated following a course of antidepressant treatment [2]. In addition to its neurotrophic effects, BDNF has proangiogenic features as well. The importance of BDNF was suggested also in high cardiovascular risk conditions, such as obesity, metabolic syndrome, and coronary atherosclerosis [3–5]. It is hypothesized to play a protective role in cardiovascular pathophysiology as its higher serum level was found to be associated with decreased risk of cardiovascular disease and mortality [6]. It was demonstrated that circulating BDNF is influenced by age and gender [7], the presence of diabetes [8], and the use of benzodiazepines in different neurological diseases [9], correlates with total cholesterol [4], and BDNF is stored and released from platelets during activation [10]. Hypertension has widely studied psychosomatic connections [11, 12]; however, the role of BDNF in this condition has not been extensively evaluated yet.

Affective temperament types (depressive, cyclothymic, hyperthymic, irritable, and anxious) are subclinical, trait-related manifestations and commonly the antecedents of minor and major mood disorders [13]. Previously, we clarified an association between dominant cyclothymic affective temperament and hypertension [14]. Recently, we demonstrated decreased seBDNF level in chronic hypertensive patients with dominant anxious, irritable, depressive or cyclothymic temperaments compared with hypertensive controls without dominant temperaments [15]. However, the association between affective temperament scores, as continuous variables and seBDNF in chronic hypertension, has not been clarified yet.

Arterial stiffening is increasingly recognized as an independent risk factor for cardiovascular diseases. Carotid–femoral pulse wave velocity (PWV) is the most accepted non-invasive arterial stiffness parameter for cardiovascular risk assessment among hypertensive patients [16]. In different animal models, BDNF was shown to be vasorelaxant not only on pulmonary arteries [17] but also on rat aortic rings [18]. Based on these data, a possible association between the seBDNF level and different arterial stiffness parameters can also be supposed in humans.

We hypothesized that as hypertension is a risk factor for cardiovascular diseases and BDNF is protective in cardiovascular pathology, seBDNF can be altered in hypertension. We also presumed that seBDNF is associated with different affective temperaments, depression, anxiety, and arterial stiffness parameters providing a new bridge of psychosomatic processes.

## Methods

In this cross-sectional study, chronic (>12 months medication) well-controlled or grade 1 consecutive hypertensive Caucasian patients (HT) and age-matched healthy controls (CONT) of three primary care practices were involved. All of the chronic hypertensive patients of our previous, pilot study [15] were involved into this study as well. Data of the involved subjects were analyzed for the relationship between the seBDNF level, routine laboratory parameters, affective temperaments, anxiety, depression, and arterial stiffness parameters. Exclusion criteria for HT were the presence of atrial fibrillation, treated depression, bipolar disorder or dementia posing an obstacle to completing questionnaires. Moderate use of the anxiolytic alprazolam (less than 0.5 mg/day) was not a restrictive criterion. In the case of CONT, the denial of consent was the only exclusion criterion.

### Evaluation of affective temperaments, depression, and anxiety

The Temperament Evaluation of Memphis, Pisa, Paris and San Diego Autoquestionnaire (TEMPS-A) was used to assess affective temperaments on depressive, cyclothymic, hyperthymic, irritable, and anxious subscales, requiring ‘yes’ (score 1) or ‘no’ (score 0) answers [19]. It contains 110 items (109 in the version for males), and the questions of the various temperament types are grouped together as follows:

1. depressive temperament: questions 1 to 21 (21 points);
2. cyclothymic temperament: questions 22 to 42 (21 points);
3. hyperthymic temperament: questions 23 to 63 (21 points);
4. irritable temperament: questions 64 to 84 (21 points in women and 20 points in men version);
5. anxious temperament: questions 85 to 110 (26 points).

The Beck depression inventory (BDI) is a 21-question multiple-choice self-report questionnaire, one of the widely used instruments for measuring the severity of depression. Participants are asked to make ratings on a four-point scale, where a higher score correlates with more severe depression [20].

Hamilton anxiety scale (HAM-A) was evaluated by the examiner to study the severity of anxiety. The scale consists of 14 items, and each item is scored on a scale of 0 (not present) to 4 (severe anxiety) [21].

### Measures of blood pressure and arterial stiffness

Arterial stiffness parameters were evaluated with the validated tonometric PulsePen device (DiaTecne, Milan, Italy). Measurements were performed in a temperature-controlled room in supine position, on the day of blood sampling, prior to it, between 7:00 and 8:00 a.m. Patients were asked to refrain from eating, smoking, and caffeine-containing drinks in the morning of the procedure, but to take the regular blood pressure medication. Upon arrival after 5 min rest, two brachial blood pressure measurements were taken on each arm in the sitting position with a validated oscillometric blood pressure device (Omron M3). The mean value of the higher side was further taken into calculation as brachial systolic (brachial SBP) and diastolic (brachial DBP) blood pressures and heart rate. Next, subjects were equipped with arterial stiffness measurement devices and then rested in the supine position for approximately 15 min before being measured. The mean of two successful measurements was used in the statistical calculations. In the PWV calculations, 80 % of the carotid–femoral distance was used following the recent guideline [22]. Augmentation index (AI), central systolic blood pressure (cSBP), central pulse pressure (cPP), and pulse pressure amplification (PPAmp) were also calculated. As PulsePen calculates pressure values using brachial diastolic blood pressure calibration, the calculated central and brachial diastolic blood pressure values were identical [23].

### Measurement of seBDNF concentration

Peripheral blood samples of patients were collected in anticoagulant-free tubes, right after the measurement of arterial stiffness. After centrifugation (3600 rpm for 6 min), the serum was stored at −20 °C. SeBDNF was measured using commercially available sandwich enzyme-linked immunosorbent assay (R&D Systems, Minneapolis MN, USA) according to the manufacturer’s protocol, and serum BDNF level was determined in pg/mL.

### Statistical analysis

Normality of the parameters was tested with the Kolmogorov–Smirnov test. Descriptive characteristics, laboratory, arterial stiffness parameters and TEMPS-A, BDI, HAM-A scores were compared between CONT and HT groups using unpaired Student’s *t* tests or Mann–Whitney rank sum test for data failing tests of normality. The equality of variances was studied with Levene’s test. Pearson correlation coefficients were calculated to study the relationship between seBDNF and all other factors measured. Hierarchic linear regression analysis was used to study the determinants of seBDNF in the whole population with a stepwise entry of variables with either previously described association with seBDNF or with a significant univariate correlation with seBDNF in the present data set. As a bidirectional association can be hypothesized between affective temperaments and hypertension [14], predetermined interaction analysis was performed to investigate moderation between hypertension and affective temperament scores on seBDNF level.

Data were expressed as mean ± standard deviation or mean with interquartile ranges. *p* < 0.05 was considered to be significant. SPSS 13.0 for Windows was used in calculations.

## Results

Altogether, 151 HT and 32 CONT subjects were involved. Eleven of the invited HT and four CONT did not give their informed consent and were excluded. Baseline demographic and laboratory parameters, current medication, TEMPS-A, BDI, and HAM-A scores, central blood pressure, and arterial stiffness parameters as well as seBDNF levels are shown in Table 1. The median number of the used antihypertensive compounds was 2 (IQR: 2–3). Differences between CONT and HT were found in body weight and BMI, serum glucose, cholesterol, LDL and HDL, BDI and HAM-A scores, in the brachial and central systolic blood pressure and the brachial pulse pressure. SeBDNF was elevated in HT (Table 1). In the analysis of simple correlations, the following parameters were found to be associated significantly with seBDNF: hypertension (*r* = 0.174, *p* = 0.018), serum cholesterol (*r* = 0.194, *p* = 0.009), LDL (*r* = 0.208, *p* = 0.015) and HDL level (*r* = 0.204, *p* = 0.006), platelet count (*r* = 0.188, *p* = 0.011), pulse pressure amplification (*r* = 0.157, *p* = 0.037), and hyperthymic temperament score (*r* = 0.189, *p* = 0.010). Tendencies of inverse correlations were found with the presence of diabetes or the use of alprazolam, but these were not significant (*r* = −0.114, *p* = 0.12 and *r* = −0.103, *p* = 0.16, respectively).

**Table 1:** Demographic, laboratory, hemodynamic, and arterial stiffness parameters; subjects’ questionnaire scores

	CONT	HT
N (male:female)	32 (12:20)	151 (58:93)
Age [year]	61.1 (55.9–70.5)	63.7 (57–71)
Duration of hypertension [year]	–	11 (5–18)
Diabetes [n (%)]	–	38 (25.2)
Cardiovascular disease [n (%)]	–	21 (13.9)
Current smoker [n (%)]	3 (9.4)	22 (14.6)
Body height [cm]	168.8 ± 8.6	166.8 ± 8.6
Body weight [kg]	72.4 ± 12.1	*79.7* *±* 14*
BMI [kg/m^2^]	24.5 ± 5.4	*28.6* *±* 4.5*
Platelet count [G/l]	239.6 (215–277)	257 (209.7–303.2)
Glucose [mmol/l]	5.36 (4.88–5.81)	6.15 (5.11–6.7)*
GFR-EPI [ml/min/1.73 m^2^]	79.7 (69.5–82.2)	77.9 (67–90)
Uric acid [µmol/l]	313.7 ± 11.6	318.4 ± 6.3
Cholesterol [mmol/l]	5.57 (4.97-6.05)	5.18 (4.37- 5.98)*
LDL [mmol/l]	3.46 ± 0.91	3.07 ± 1.04
HDL [mmol/l]	1.68 (1.31–1.98)	1.40 (1.15–1.61)*
Triglyceride [mmol/l]	1.16 (0.75–1.43)	1.67 (1.08–2.06)*
Regular medication [n (%)]		
ACE inhibitors	–	93 (61.5)
ARBs	–	34 (22.5)
CCBs	–	67 (44.4)
Beta blockers	–	87 (57.6)
Diuretics	–	68 (45)
Antiplatelet drugs	–	44 (29.1)
Statins	5 (15.7)	54 (35.7)
Alprazolam	–	23 (15.2)
TEMPS-A		
Depressive	5.9 (4–7)	7.1 (5–9)
Cyclothymic	2.9 (0–4)	3.9 (1–6)
Hyperthymic	11.2 ± 4	11 (4.2)
Irritable	3.2 (2–4)	4.3 (2–6)
Anxious	4.1 (1–6)	6.3 (2–9)
BDI	2.8 (1–4)	6.3 (3–9)*
HAM-A	3.9 (1–6)	7.4 (2–10)*
Heart rate [1/min]	72.1 (66.6–78.2)	72.7 (64.1–77.2)
Brachial SBP [Hgmm]	125.5 ± 9.3	*133.0* *±* 12.3*
Brachial DBP [Hgmm]	72 ± 6.4	75 ± 9
Brachial PP [Hgmm]	51.5 (46.4–56.7)	56.7 (46.4–63)*
Central SBP [Hgmm]	117 (111.2–122.3)	124.1 (113.4–131.6)*
Central DBP [Hgmm]	67.1 ± 7	69.8 ± 8.2
Central PP [Hgmm]	49.9 (43.2–54.5)	54.3 (45.2–61)
PP amplification	1.08 (1.03–1.14)	1.07 (0.98–1.12)
PWV [m/sec]	8.6 (7.4–9.2)	9.3 (7.8–10)
AIx (%)	13.2 (5.75–23)	17.8 (8.5–25.1)
Serum BDNF (pg/ml)	21202.6 ± 6045.5	*24880* *±* 8279*

Continuous data are presented as mean (SD) or mean (interquartile range)

Categorical parameters are presented as % (n)

*BMI* body mass index, *ARBs* angiotensin II receptor blockers, *CCBs* calcium channel blockers, *SBP* systolic blood pressure, *DBP* diastolic blood pressure, *PP* pulse pressure, *PWV* pulse wave velocity, *AIx* augmentation index, *TEMPS-A* Temperament Evaluation of Memphis, Pisa, Paris and San Diego Autoquestionnaire, *BDI* Beck depression inventory, *HAM-A* Hamilton anxiety scale

^*^ *p* < 0.05

**Fig. 1:**
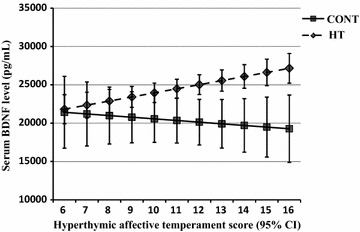
Association between serum BDNF level and hyperthymic affective temperament score in hypertensive patients (HT) and in controls (CONT). *Continuous line* with *squares* represents CONT, while *broken line* with rhombs represents HT

Table 2 demonstrates the results of hierarchical linear regression models. In the final model adjusted for all potential confounders, one unit increase in the hyperthymic score was associated with a 405.8 pg/mL higher seBDNF and the presence of hypertension with a 6121.2 pg/mL higher seBDNF. We found an interaction (*p* = 0.002) between hypertension and hyperthymic temperament score on seBDNF in the whole study population: there was no significant association between hyperthymic score and seBDNF in CONT (*p* = 0.545) and a unit increase in hyperthymic score was associated with a 533.3 (95 %CI 241.3–825.3) pg/mL higher seBDNF level in HT (*p* < 0.001). The different impact of hyperthymic score in seBDNF in HT and CONT is shown in Fig. 1.

**Table 2:** The predictive values of hypertension and hyperthymic affective temperament score on serum BDNF level in different models evaluated with linear regression analysis in the whole study population (*n* = 183)

Models	Beta	Std. Error	Std. Beta	*p*	R^2^
Model 1					0.036
Hypertension	4149.6	1681.8	0.190	0.015	
Model 2					0.050
Hyp. temp. score	410.7	140	0.225	0.004	
Model 3					
Hypertension + hyp. temp. score					0.089
Hypertension	4310.3	1640.6	0.198	0.009	
Hyp. temp. score	422.2	137.6	0.231	0.003	
Model 4: Model 3 + age + sex					0.107
Hypertension	4365.1	1636.7	0.200	0.008	
Hyp. temp. score	449.8	138.1	0.246	>0.001	
Model 5: Model 4 + diabetes					0.132
Hypertension	5161.4	1661.2	0.237	0.002	
Hyp. temp. score	459.4	136.6	0.251	>0.001	
Model 6: Model 5 + cholesterol + HDL					0.158
Hypertension	5954.7	1685.5	0.273	>0.001	
Hyp. temp. score	430.6	136.8	0.235	0.002	
Model 7: Model 6 + platelet number					0.176
Hypertension	5540	1688.4	0.254	>0.001	
Hyp. temp. score	431.9	135.8	0.236	0.002	
Model 8: Model 7 + BDI + HAM-A + Alp.					0.191
Hypertension	6167	1748.4	0.283	>0.001	
Hyp. temp. score	408	140.9	0.223	0.004	
Model 9: Model 8 + PPamp.					0.202
Hypertension	6121.2	1742.3	0.281	>0.001	
Hyp. temp. score	405.8	140.4	0.222	0.004	

*Hyp. temp. score* hyperthymic affective temperament score, *BDI* Beck depression inventory, *HAM-A* Hamilton anxiety scale, *Alp* patients regularly using alprazolam, *PPamp* pulse pressure amplification

## Discussion

Here, we demonstrated for the first time in the literature that in chronic hypertensive patients, seBDNF is elevated, and hyperthymic affective temperament score and the presence of hypertension are independent determinants of seBDNF level. In hypertensive patients, the elevation of hyperthymic temperament score is associated with the elevation of seBDNF; however, this association is not present in healthy subjects.

We suppose that the observed BDNF elevation in HT can be part of a protective compensatory mechanism targeting peripheral neurons and vascular cells. BDNF has beneficial effects on the regulation of blood pressure, as it is involved not only in the development, but also in the survival of arterial baroreceptor system [24]. Vascular endothelial cells are proved to produce BDNF [25]. In patients with angina pectoris, Jiang et al. demonstrated that low plasma BDNF level was associated with a higher probability of major cardiovascular events than a middle level or a high level during the 4-year follow-up period [26]. Moreover, in a recently published population-based study, higher seBDNF was found to be associated with decreased risk of cardiovascular morbidity and mortality [6]. On the contrary, decreased serum BDNF was found to be associated with increased risk of incident stroke/TIA [27]. In our study, the positive correlation with HDL and also with pulse pressure amplification, where higher values refer to better vascular conditions [28], also supports the plausible beneficial effect of BDNF in hypertension.

Some of the findings of our study were already described in the literature, such as the seBDNF correlation with cholesterol and LDL [4], as well as with platelets [10]. As stored BDNF is released from platelets during clotting [10] and in essential hypertension, increased platelet activation is a trigger of hypercoagulable state [29], our finding that platelet count is positively correlated with seBDNF may refer to a chief source of seBDNF in this pathological condition.

Another main finding of our study is that hyperthymic affective temperament is an independent determinant of seBDNF. This temperament is characterized by exuberant, upbeat, overenergetic, and overconfident lifelong traits [30]. We suppose that patients with higher hyperthymic temperament scores might have reduced inclination to cardiovascular complications, due to the beneficial effect of elevated seBDNF, a hypothesis that needs to be confirmed in follow-up studies. As the observed association between hyperthymic temperament score and seBDNF was only present in our hypertensive patients, we suppose an active role of affective temperaments not only in psychiatric but also in cardiovascular pathophysiology.

Interestingly, in our study, no association of seBDNF with anxiety or depression was found. We suppose that this phenomenon can be explained by the mild anxiety and depression severity of HT patients.

In contrast to the literature, the presence of diabetes or the use of the benzodiazepine alprazolam was not significantly correlated with seBDNF; however, the direction of correlations was as expected. We think that in both cases, the lack of significance was caused by the low proportion of diabetic or alprazolam user patients in our cohort.

The associations between seBDNF level and arterial stiffness parameters have never been evaluated in any patient population yet. Since BDNF has a relaxant effect on pulmonary arterial and aortic rings in different animal models [17, 18], we supposed a possible link between BDNF and arterial stiffness parameters. In contrast to this, in our study seBDNF showed an association only with pulse pressure amplification, but even this failed to be an independent predictor in regression analysis. We suppose from these findings that seBDNF may exert its protective role rather on the level of the endothelium and perivascular nerves than on the level of large arteries.

The main limitation of our study comes from its cross-sectional design which precludes causal inference. In addition, the number of the subjects involved into the study limited the number of potential confounding variables that were involved in the final regression model. Consequently, the presence of sleeping disorder, the amount of alcohol intake or the habit of regular exercise, variables with documented influence on BDNF level, were not involved into the final analysis (these were not significantly correlated with seBDNF in univariate models, data are not shown). Moreover, other potential confounders, like childhood trauma, stress or sunlight exposition were not evaluated. In addition to these limitations, although we used standardized questionnaires and excluded patients with dementia, a complete exclusion of misinterpretations or mistakes by the patients is impossible.

## Conclusions

In conclusions, our results suggest a complex psychosomatic involvement of BDNF in the pathophysiology of hypertension, where hyperthymic affective temperament may have a protective role. The impact of this phenomenon for cardiovascular outcome has to be clarified in prospective studies, but its mechanism is probably not mediated by large arteries.

## Abbreviations

AC: abdominal circumference
AI: augmentation index
ARBs: angiotensin II receptor blockers
BDI: Beck depression inventory
BDNF: brain-derived neurotrophic factor
BMI: body mass index
Brachial DBP: brachial diastolic blood pressure
Brachial PP: brachial pulse pressure
Brachial SBP: brachial systolic blood pressure
Central DBP: central diastolic blood pressure
Central MBP: central mean blood pressure
Central PP: central pulse pressure
Central SBP: central systolic blood pressure
CKD-EPI GFR: glomerular filtration rate assessed by the chronic kidney disease epidemiology collaboration glomerular filtration rate equation
HAM-A: Hamilton anxiety scale
HR: heart rate
Hyp. temp. score: hyperthymic affective temperament score
PP amplification: pulse pressure amplification
PWV: carotid–femoral pulse wave velocity
seBDNF: serum brain-derived neurotrophic factor
TEMPS-A: the Temperament Evaluation of Memphis Pisa, Paris and San Diego questionnaire
